# Cell fusing agent virus and dengue virus mutually interact in *Aedes aegypti* cell lines

**DOI:** 10.1038/s41598-017-07279-5

**Published:** 2017-07-31

**Authors:** Guangmei Zhang, Sultan Asad, Alexander A. Khromykh, Sassan Asgari

**Affiliations:** 1 Australian Infectious Disease Research Centre, School of Biological Sciences, The University of Queensland, Brisbane, QLD 4072 Australia; 2 Australian Infectious Disease Research Centre, School of Chemistry and Molecular Biosciences, The University of Queensland, Brisbane, QLD 4072 Australia

## Abstract

The genus *Flavivirus* contains more than 70 single-stranded, positive-sense arthropod-borne RNA viruses. Some flaviviruses are particularly medically important to humans and other vertebrates including dengue virus (DENV), West Nile virus, and yellow fever virus. These viruses are transmitted to vertebrates by mosquitoes and other arthropod species. Mosquitoes are also infected by insect-specific flaviviruses (ISFs) that do not appear to be infective to vertebrates. Cell fusing agent virus (CFAV) was the first described ISF, which was discovered in an *Aedes aegypti* cell culture. We found that while CFAV infection could be significantly reduced by application of RNAi against the *NS5* gene, removal of the treatment led to quick restoration of CFAV replication. Interestingly, we found that CFAV infection significantly enhanced replication of DENV, and vice versa, DENV infection significantly enhanced replication of CFAV in mosquito cells. We have shown that CFAV infection leads to increase in the expression of ribonuclease kappa (RNASEK), which is known to promote infection of viruses that rely on endocytosis and pH-dependent entry. Knockdown of RNASEK by dsRNA resulted in reduced DENV replication. Thus, increased expression of RNASEK induced by CFAV is likely to contribute to enhanced DENV replication in CFAV-infected cells.

## Introduction

Flaviviruses have single-stranded positive sense RNA genomes and are transmitted to vertebrate species mostly by mosquitoes and other arthropods^1^. A number of these viruses cause serious diseases leading to considerable morbidity and mortality around the world. Among mosquito-borne flaviviruses are dengue virus (DENV), West Nile virus, Japanese encephalitis virus and yellow fever virus. Because of poor vector control and lack of effective vaccines or drugs, the resurgence and expansion of mosquito-borne diseases has been an important global health concern in recent decades; for example dengue and Zika, which are most commonly transmitted by the mosquito *Aedes aegypti* ^2^.

Mosquitoes can also become infected by insect-specific flaviviruses (ISFs) that do not infect vertebrates. Cell fusing agent virus (CFAV) was the first described ISF discovered from an *Ae. aegypti* cell line^3^. It was later reported from *Ae. aegypti* mosquitoes in Puerto Rico^4^. It is believed that this virus is transmitted vertically^4^ and therefore the embryos used to initiate the original cell line must have been infected. CFAV has similar genome size, structure and gene order to other flaviviruses. For example, there is over 40% identity of the amino acid sequence of the NS5 protein between CFAV and other flaviviruses^5^. It has been reported that segments of the CFAV genome have integrated into *Ae. aegypti* and *Ae. albopictus* genomes^6^, which suggests that CFAV has been persistently infecting the mosquitoes for a long time. However, it is unclear what functional role the CFAV plays in mosquitoes.

While coinfections or superinfections (sequential infections) of a variety of homologous or heterologous arboviruses has been tested in different insect cell lines (mostly in C6/36 cells) and mosquitoes (reviewed in refs 7 and 8), none of these studies included CFAV. In these studies, the outcomes of coinfections or superinfections were either negative or no interference. In this study, we investigated infection of *Ae. aegypti* cell lines, Aag2 and Aa20, with CFAV and explored the interaction of CFAV with DENV.

## Materials and Methods

### Insect cell lines

**Figure 1:**
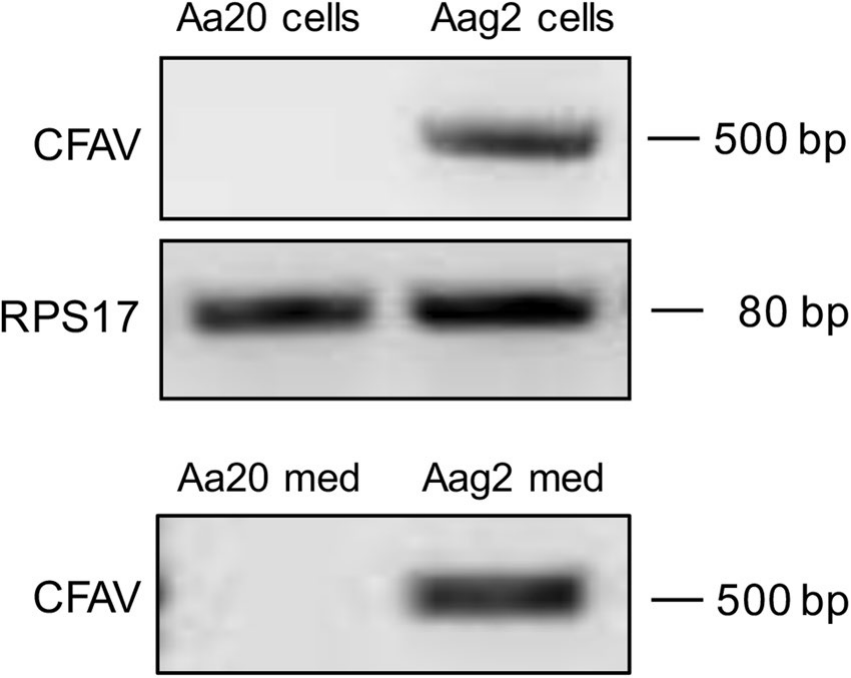
CFAV is found in *Ae. aegypti* Aag2 cell line. RT-PCR analysis of RNA extracted from *Ae. aegypti* Aa20 and Aag2 cells, and their corresponding media (med). *RPS17* gene was used as control. Full-length gel images are displayed in the Supplementary Information file.

*Ae. aegypti* Aag2 cells were maintained in a medium with a 1:1 mixture of Mitsuhashi-Maramorosch and Schneider’s insect media (Invitrogen) supplemented with 10% FBS and in the presence of penicillin (100 U ml^−1^) and streptomycin (100 μg ml^−1^). Aa20 cells established from *Ae. aegypti* larvae were kindly provided by the late Prof Richard Elliott. The cells were maintained in Leibovitz’s L15 medium supplemented with 10% FBS and 10% Tryptose phosphate broth^9^. To infect Aa20 cells with CFAV, Aag2 culture medium containing the virus (Fig. 1) was collected, centrifuged at 2150× g for 5 min to remove cells and debris, and used as a CFAV inoculation source. The titre of CFAV was determined using tissue culture infectious dose_50_ (TCID_50_) endpoint fixed cell-enzyme-linked immunosorbent assay method as previously described^10^.

### RNA extraction, cDNA synthesis and polymerase chain reaction (PCR)

Total RNA from mosquito cells was isolated using Tri-Reagent (Ambion Inc., USA) after removal of medium and washing cells three times with PBS. RNA was incubated with DNase I at 37 °C for 10 min and then inactivated at 75 °C for 10 min. The first strand cDNA was synthesized by reverse transcription (RT) with CFAV-specific or poly(dT) primers (for ribosomal protein S17, *RPS17*, housekeeping gene detection as control). In each RT reaction, approximately 2 µg of total RNA was used as template in a volume of 20 μl. Following cDNA synthesis, 2 μl of RT products was used for each PCR in a total reaction volume of 25 μl with *CFAV NS5* gene-specific primers (Forward: 5′-GCCCACATCTGGGCRTRNGCCTTNGC-3′; Reverse: 5′-GGGCAAGTARBMACTTATGCVTTGAACAC-3′). These are referred to as CFAV-specific detection primers. Amplification was performed at 95 °C for 1 min, followed by 35 cycles of 95 °C for 30 sec, 56 °C for 30 sec, 68 °C for 1 min, and a final extension at 68 °C for 5 min. PCR products were run on agarose gels, stained by ethidium bromide, and bands were visualized in a gel documentation system (Red, Proteinsimple) using UV light. Images were recorded and shown in negative.

### RT-qPCR

Total RNA was extracted from mosquito cells and treated with DNase I. The synthesis of first strand cDNA was carried out using a specific reverse primer to DENV or CFAV (CFAV-qR 5′-CACAACGGTAGCGAGAGACA-3′). Following the RT, qPCRs with DENV (forward: 5′-GTGGTGGTGACTGAGGACTG-3′; reverse: 5′-CCATCCCGTACCAGCATCCG-3′) and CFAV specific primers (CFAV-qF 5′-CTGATGTGCGTGCAGTTCTT-3′ and CFAV-qR) were carried out to determine the DENV and CFAV genomic RNA levels in cells. Platinum SYBR Green Mix (Invitrogen) was used for qPCR with 1 μl of RT products in a Rotor-Gene thermal cycler (QIAGEN) as described above. The *RPS17* gene was used for normalizing data as described previously^11^. Each reaction was run with 3 biological replicates, each with 3 technical replicates. The relative abundance of viral RNA to the host reference gene was determined by qGENE software and analyzed using GraphPad Prism.

### RNAi-mediated silencing

For RNAi-based experiments, dsRNAs were synthesized *in vitro* using the T7 MEGAscript transcription kit according to the manufacturer’s instruction (Ambion Inc., USA). T7 promoter sequences (TAATACGACTCACTATAGGG) were incorporated in both forward and reverse primers designed to amplify an around 500 bp fragment of the *CFAV NS5* (forward: 5′-GAGGAGGATCTGGAGGATGA-3′; reverse: 5′-CCCTCGCCACCTGTACCTTA-3′). These are referred to as CFAV RNAi primers, which were different to the CFAV-specific detection primers. For dsRNA synthesis, 200–500 ng of PCR product were used for each reaction. Reaction was incubated at 37 °C overnight, DNase-treated and precipitated by the lithium chloride method following the manufacturer’s instruction. A total of 4 μg of dsRNA was used to transfect Aag2 cells with 5 μl of Cellfectin transfection reagent (Invitrogen). The cells were transfected again with the same reagent at 48 h intervals after the first transfection. Cells were collected for RNA extraction as required for further analysis at 24 h after the second transfection. Gene silencing was confirmed by RT-PCR using CFAV-specific detection primers.

RNASEK (accession no. XM_001663743.1) was silenced as described above using forward (5′-CTATATCCATAGTGTGGCGC-3′) and reverse (5′-AGCAACAGTTGTGCGACTGT-3′) primers with T7 promoter sequences incorporated in them. Silencing of the gene was confirmed by qPCR forward (5′-CCGATCTGTGGACCCAAACT-3′) and reverse (5′-GAAGACACCCATCAGGAGCA-3′) primers, outside the dsRNA region.

### In-cell Western plaque assay

Vero cells (an African green monkey cell line) were seeded into a 96-well plate and incubated at 37 °C overnight until 90–100% confluent. Medium from DENV-infected cells was diluted into four dilutions: 10^0^, 10^−1^, 10^−2^, 10^−3^. Fifty µl of virus solution was added into each well and incubated at room temperature for 1 h, then incubated at 37 °C for an additional hour. The inoculum was removed, and cells were overlaid with 47.5% medium and 47.5% of carboxymethyl cellulose gel supplemented with 4% FBS. After 72 h incubation at 37 °C, gels were discarded and cells were fixed with ice cold 80% acetone in PBS at −20 °C for 20 min. Plate was dried fully. Fifty µl 5% skin milk in PBST as blocking solution was added to each well and incubated at 37 °C for 0.5 h. Fifty µl of first antibody (anti-DENV2-Envelope protein antibody 1:1000 dilution in PBST) was added into each well and incubated at 4 °C for 2 h. Then, cells were washed three times with PBS containing 0.05% Tween 20. Secondary IR dye conjugated antibody (Sigma) was added (1:2500 dilution), and incubated at 37 °C for 1.0 h. Cells were washed three times with PBS containing 0.05% Tween 20. Plate was scanned using LI-COR Biosciences Odyssey Infrared Imaging System and plaques were counted.

### Statistical analysis

Unpaired t-test was used to compare differences between two individual groups, while one-way ANOVA with Tukey’s post-hoc test was carried out to compare differences between more than two groups. Data that did not pass the normality test were re-analysed by the non-parametric Wilcoxon test indicated in their relevant figure legends.

## Results

### CFAV was found in *Ae. aegypti* Aag2 cells, but not in Aa20 cells

**Figure 2:**
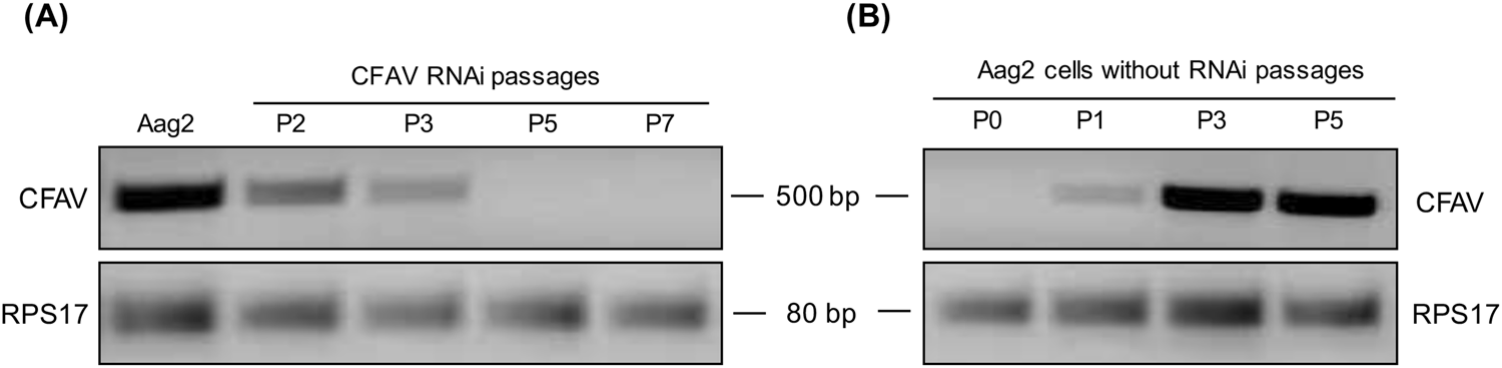
CFAV replication is suppressed by NS5 RNAi in Aag2 cells, but can come back without dsRNA application. RNAi-mediated silencing of CFAV *NS5* gene was carried out in Aag2 cells for 72 h. (**A**) RT-PCR analysis of CFAV was performed using RNA extracted from Aag2 and Aag2 cells transfected with NS5 dsRNAs. **(B)** RT-PCR analysis of CFAV was performed by using RNA extracted from the Aag2 cells transfected with NS5 dsRNA for 5 passages and then cultured normally with complete medium for 5 passages. *RPS17* gene was used as control to show the integrity of RNA. P refers to passage number. Full-length gel images are displayed in the Supplementary Information file.

Cell fusing agent virus (CFAV), from the genus *Flavivirus*, was originally isolated from an *Ae. aegypti* cell line (Peleg) in laboratory^3^. In contrast to the majority of known flaviviruses, CFAV is an ISF and does not have a vertebrate host^3^. Firstly, we investigated the presence of CFAV in *Ae. aegypti* cell lines available to us by RT-PCR with CFAV-specific detection primers. Results showed that CFAV RNA was present in Aag2 cells as previously reported^12^, but not in Aa20 cells (Fig. 1). Consistently, CFAV was only detected in the medium from Aag2 cells and not in that of Aa20 cells (Fig. 1). Because Aag2 is one of the most commonly used mosquito cell lines and that existing CFAV infection might affect the physiology and transcriptional profiling of the cells, we attempted to remove CFAV from Aag2 cells by transfecting them with dsRNA to CFAV *NS5*. After using *NS5* dsRNA for five passages in Aag2 cells, RT-PCR results showed that CFAV RNA was hardly detectable (Fig. 2A). However, when the *NS5* dsRNA transfection was stopped, CFAV RNA could be detected again already in the first passage, and its levels recovered as quickly as by passage 3 to the level observed in the original Aag2 cells (Fig. 2B).

### CFAV promotes dengue virus infection in mosquito cells

**Figure 3:**
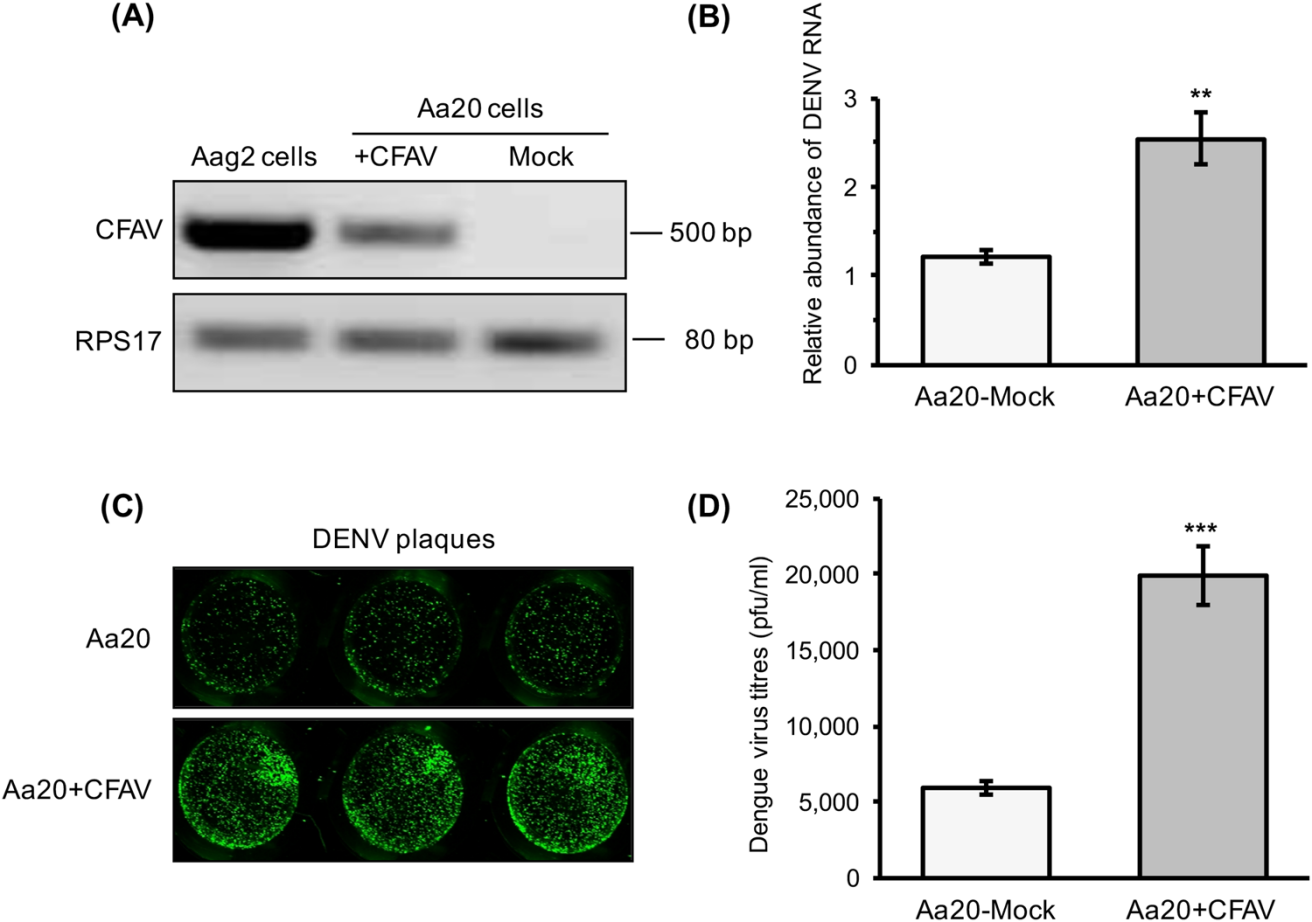
DENV replication is enhanced by CFAV in Aa20 cells. (**A**) RT-PCR analysis of CFAV was performed using RNA extracted from Aa20 mock and Aa20 cells infected with CFAV. *RPS17* gene was used as control to show the integrity of RNA. Full-length gel images are displayed in the Supplementary Information file. (**B**) RT-qPCR analysis of DENV was performed using RNA extracted from Aa20 and Aa20 + CFAV cells infected for 3 days with 1 MOI of DENV. Three biological replicates with three technical replicates were carried out for each transfection (*P < 0.05; t test). (**C**) Plaque assay of media collected from the experiment described in (**B**) without dilution using anti-DENV2-Envelope protein antibody. Each well represents a biological replicate, and only one representative replicate of the plaque assay is shown here. (**D**) DENV titration of medium from the experiment described in (**B**) (***P < 0.001; t test).

**Figure 4:**
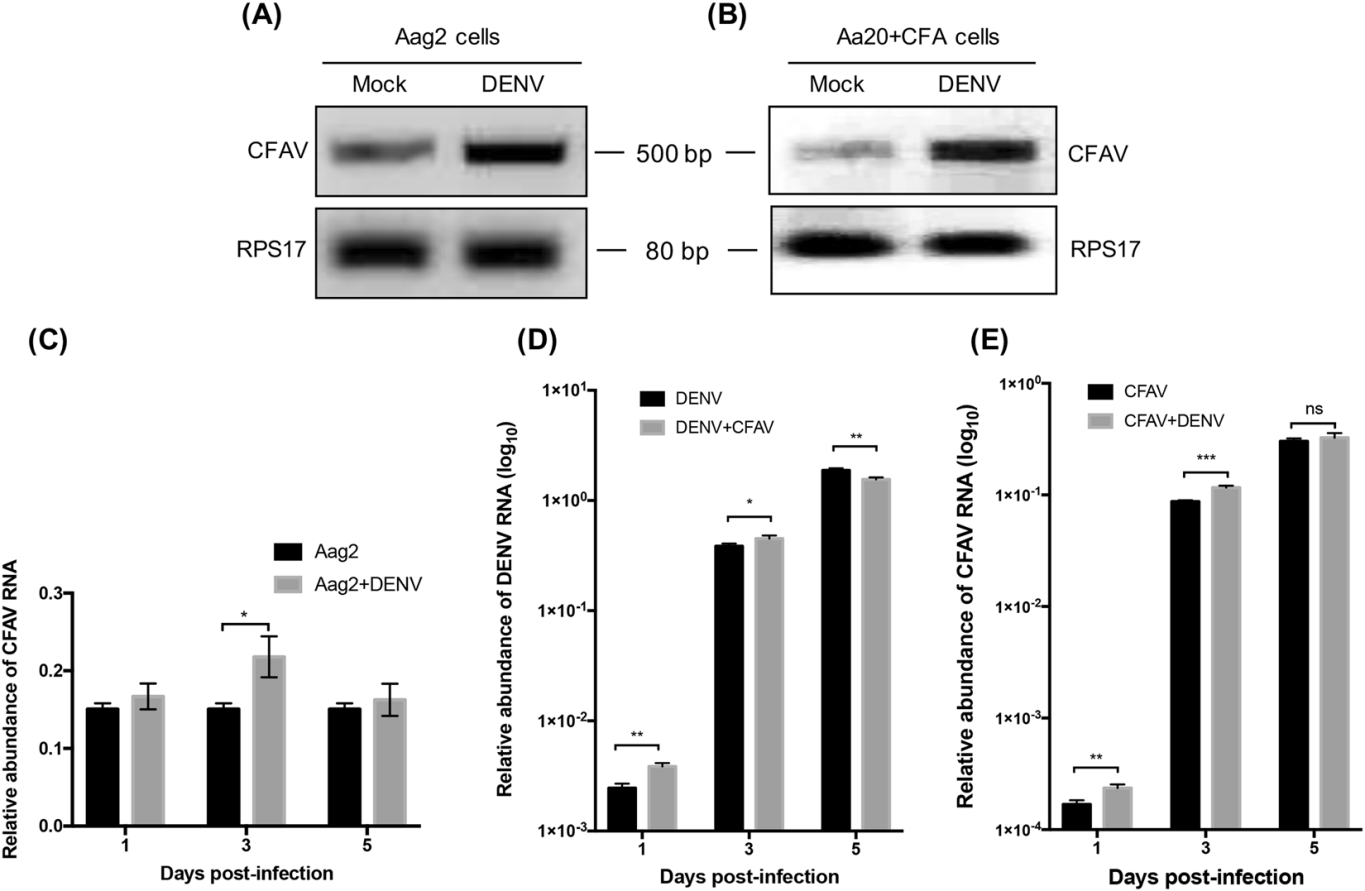
CFAV replication is increased by DENV in CFAV-infected Aa20 and Aag2 cells. (**A**) RT-PCR analysis of CFAV was performed using RNA extracted from Aag2 (Mock) and Aag2 cells infected with 1 MOI DENV for 72 h. **(B)** RT-PCR analysis of CFAV was performed by using RNA extracted from Aa20 + CFAV cells (infected with CFAV for 7 days prior to DENV infection) extracted at 72 h after infection with 1 MOI DENV. Mock represents RNA from Aa20 + CFAV cells that were not infected with DENV and collected at the same time as DENV-infected cells. *RPS17* gene was used as control to show the integrity of RNA. Full-length gel images are displayed in the Supplementary Information file. **(C)** RT-qPCR analysis of RNA from Aag2 cells infected with 0.1 MOI of DENV-2 and collected at three time points following infection. **(D)** and **(E)** RT-qPCR analysis of RNA from Aa20 cells either singly infected or co-infected with 0.1 MOI of DENV and CFAV and collected at three times points following infection, which were analysed by DENV and CFAV specific qPCR primers, respectively. *P < 0.05; **P < 0.01; ***P < 0.001; ANOVA test with Tukey *post hoc* comparison).

To determine whether prior infection of mosquito cells with CFAV may affect subsequent infection by DENV, Aa20 CFAV-free cells were infected with CFAV, collected from supernatant of cultured Aag2 cells persistently infected with CFAV. We confirmed infection of Aa20 cells with CFAV (Aa20 + CFAV cells) seven days after the inoculation using CFAV-specific detection primers (Fig. 3A). The result also confirmed that Aag2 cells produce infectious CFAV particles and that detection of CFAV RNA in the cells correlates with virus production (also see Fig. 4E which shows active replication of CFAV in Aa20 cells). Subsequently, Aa20 and Aa20 + CFAV cells were infected with MOI 1 of DENV-2 and total RNA was extracted at 72 h after infection and analysed by RT-qPCR with gene-specific primers to DENV-2. RT-qPCR results revealed that the relative abundance of DENV RNA was significantly higher in Aa20 + CFAV cells as compared with Aa20 cells (Fig. 3B). In addition, we carried out a plaque assay using the medium collected from the infected cells at 72 h post infection, which showed significantly higher DENV-2 titers in Aa20 + CFAV cells as compared with Aa20 cells (Fig. 3C and D).

### DENV enhances replication of CFAV in mosquito cells

The above results showed that CFAV promoted the replication of DENV in cells. To investigate whether DENV reversely influences the replication of CFAV, Aag2 cells and Aa20 + CFAV cells were inoculated with DENV. Total RNA at 72 hpi was extracted and analysed by RT-PCR with CFAV-specific detection primers. RT-PCR results showed that the CFAV RNA levels were higher in Aag2 cells infected with DENV as compared with mock-infected Aag2 cells (Fig. 4A). Similarly, in Aa20 + CFAV cells that were used at 7 days after CFAV infection, the CFAV RNA levels were found to be higher at 3 days following DENV infection as compared with DENV non-infected Aa20 + CFAV cells (Fig. 4B). The results suggest that DENV mutually enhances replication of CFAV.

**Figure 5:**
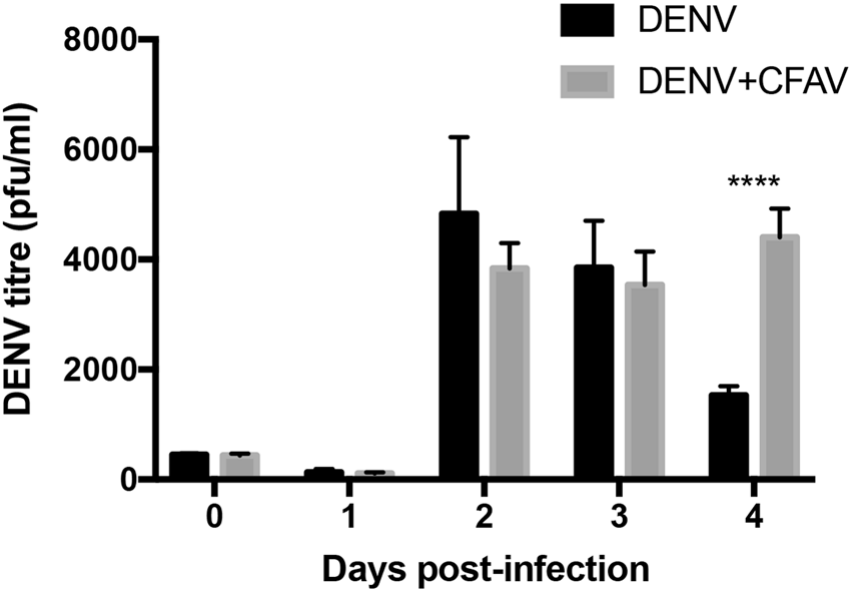
DENV virion production is enhanced by CFAV co-infection. Aa20 cells were infected with 0.1 MOI DENV only or co-infected with 0.1 MOI DENV + CFAV. Media were collected from day 0 to 4 days post-infection and analysed for virion production by plaque assay (****P < 0.0001; ANOVA test with Tukey *post hoc* comparison).

We also examined the dynamics of the effect of DENV infection on CFAV and vice versa. For this, Aag2 cells that are already infected with CFAV were infected with MOI 0.1 of DENV. RNA was extracted from cells at 1, 3 and 5 dpi of DENV and analysed by RT-qPCR using specific primers to CFAV. While in mock-infected Aag2 cells CFAV levels remained constant, in those infected with DENV, there was an increase in CFAV levels, although this was only statistically significant at 3 dpi (Fig. 4C), consistent with Fig. 4A. To examine the effect of CFAV on DENV infection, Aa20 cells were infected either with DENV alone or DENV together with CFAV (both at 0.1 MOI). RT-qPCR results showed significant but modest increase in the levels of DENV RNA in Aa20 cells when co-infected with CFAV at 1 and 3 dpi, while it decreased at 5 days post-infection (Fig. 4D). It appears that when Aa20 cells were already infected with CFAV prior to DENV infection, the effect on DENV was more prominent (Fig. 3) as compared to when they were co-infected with both viruses at the same time. Similarly, co-infection of Aa20 cells with CFAV and DENV enhanced CFAV replication as compared to CFAV infection alone (Fig. 4E). In an independent experiment, in which Aa20 cells were infected either with DENV alone or co-infected with CFAV, more DENV virions were found in the media collected from cells with CFAV-DENV co-infection at 4 dpi (Fig. 5).

### Increase in RNASEK by CFAV and its effect on DENV

**Figure 6:**
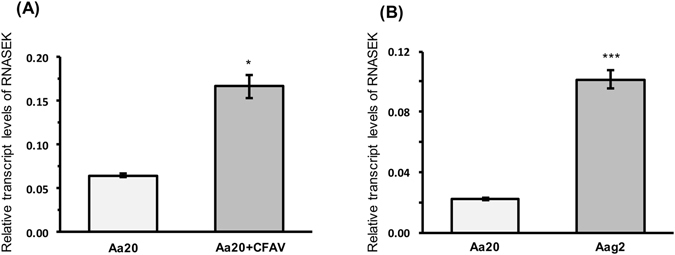
RNASEK transcript levels increased by CFAV. (**A**) RT-qPCR analysis of RNASEK was performed using RNA extracted from Aa20 and Aa20 + CFAV cells (*P < 0.0453; Wilcoxon non-parametric test). **(B)** Relative transcript levels of RNASEK in Aa20 (without CFAV) and Aag2 (infected with CFAV) cells. Three biological replicates with three technical replicates were carried out for each treatment (***P < 0.001; t test).

**Figure 7:**
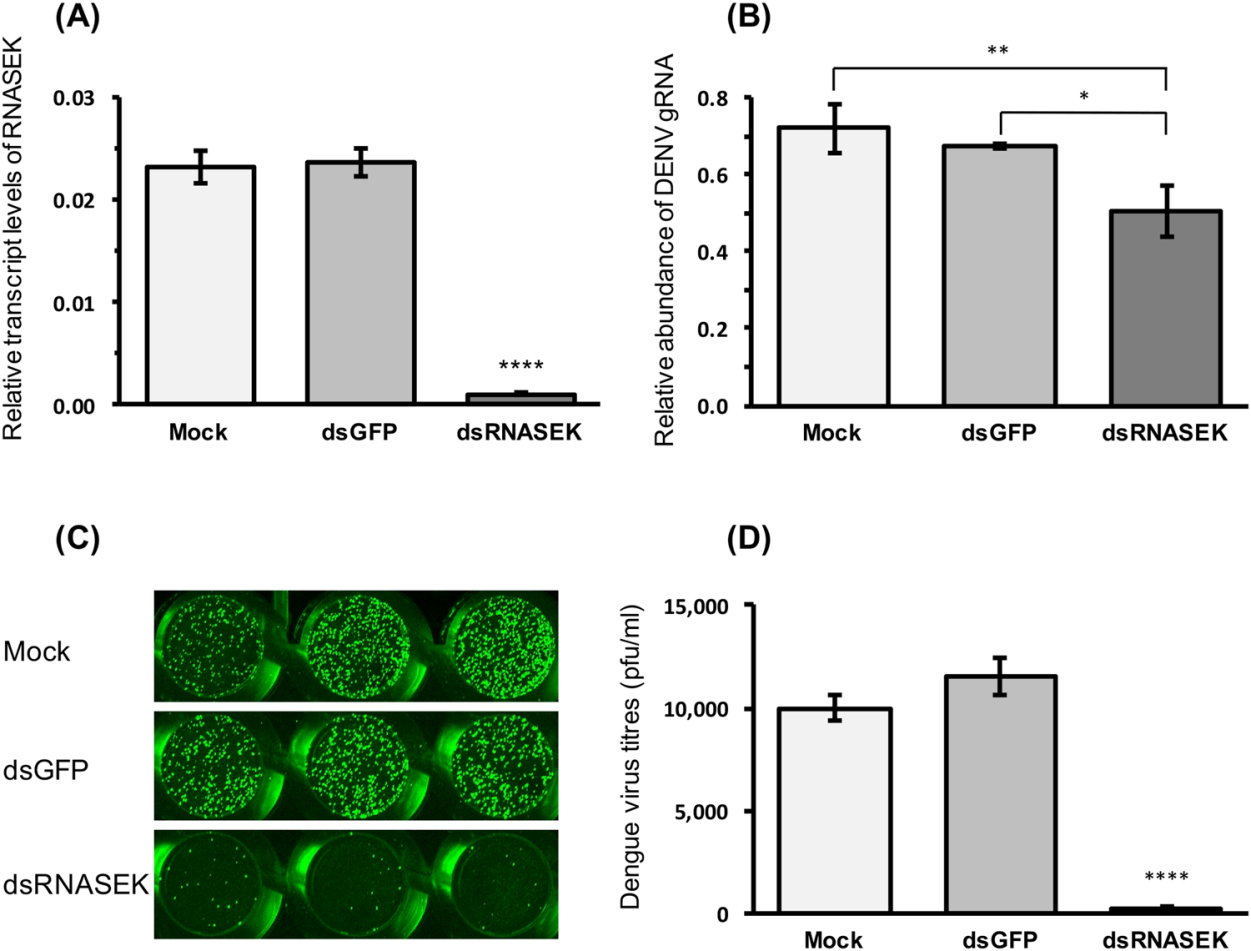
Silencing RNASEK reduces DENV replication. (**A**) Confirmation of RNASEK silencing in Aa20 cells by RT-qPCR (****P < 0.00001; ANOVA test). (**B**) Relative abundance of DENV gRNA was assessed in Aa20 cells transfected with mock, dsGFP and dsRNASEK by RT-qPCR. Three biological replicates with three technical replicates were carried out for each treatment (**P < 0.001; *P < 0.05; Wilcoxon non-parametric test with Tukey *post hoc* comparison). **(C)** Plaque assay of media collected from the experiment described in (**B**) without dilution using anti-DENV2-Envelope protein antibody. Each well represents a biological replicate, and only one representative replicate of the plaque assay is shown here. **(D)** DENV titration of medium from the experiment described in (**B**) (****P < 0.0001; ANOVA test).

In testing a number of genes (five genes cecropin, defensin, attacin, domeless and RNASEK) reported in the literature to be involved in insect virus-host interactions and immunity^13–18^, we noticed a significant increase in the transcript levels of RNASEK in Aa20 + CFAV cells as compared to Aa20 cells (Fig. 6A). The genes were assessed based on their transcriptional changes in response to DENV infection analysed by RT-qPCR. RNASEK was also highly expressed in Aag2 cells as compared to Aa20 cells that are devoid of CFAV (Fig. 6B). Ribonuclease kappa (RNASEK) was shown to promote infection of a range of viruses that rely on endocytosis and pH-dependent entry, including DENV^16^. The role of RNASEK in replication of these viruses was examined in *Drosophila* S2 cells and human osteosarcoma cells (U2OS) but not in mosquito cells. To confirm that RNASEK promotes DENV replication in mosquito cells, we silenced RNASEK in Aa20 cells by transfection of dsRNASEK into the cells (Fig. 7A) followed by their infection with 1 MOI of DENV-2. After 72 h, RT-qPCR analysis revealed significantly less DENV gRNA present in dsRNASEK-transfected cells as compared with mock or dsGFP transfected cells (Fig. 7B). This result was further confirmed by plaque assay using media collected from cells in the silencing experiment, which clearly showed substantially less infectious DENV in the media from cells transfected with dsRNASEK as compared with the controls (Fig. 7C and D). The data show that RNASEK knockdown results in reduction of DENV RNA in infected cells and of infectious virus in the culture fluid.

## Discussion

With the utilization of advanced next generation sequencing approaches for viral detection/discovery, there has been a significant increase in the isolation and characterization of ISFs recently^19–27^. The existence of ISFs in mosquitoes may affect their vector competence in transmission of pathogenic arboviruses to vertebrate hosts. For instance, in *Culex pipiens*, Culex flavivirus (CxFV) suppressed dissemination of West Nile virus (WNV) at early stages of infection (7 days), which may consequently affect the severity of enzootic transmission of WNV by the mosquito^28, 29^. In addition, it was reported that CxFV from Guatemala could significantly enhance the transmission rate of WNV in *Culex quinquefasciatus* from Honduras^30^. Similarly, another ISF, Palm Creek virus, repressed replication of WNV and Murray Valley encephalitis virus in mosquito cells, but did not repress replication of the alphavirus Ross River virus^27^. Further, Eilat virus, reduced replication of several mosquito-borne viruses such as Sindbis virus, eastern, western, and Venezuelan equine encephalitis viruses, and Chikungunya virus^26^. While the majority of examples indicate that ISFs may have a negative effect on the replication of pathogenic flaviviruses, we found rather positive interaction of CFAV and DENV; although as mentioned above in the case of CxFV and WNV in *Cx. quinquefasciatus*, our observation is not the first instance of a positive interaction between an ISF and a pathogenic flavivirus.

Cell fusing agent virus (CFAV) was the first described ISF, which was discovered in an *Ae. aegypti* cell culture named Peleg^3^. Different strains of the virus have also been detected from field-collected *Ae. aegypti* and other mosquito species^4, 21, 31, 32^ CFAV has similar genome size, structure and gene order to other flaviviruses^33^. In the present study, CFAV was detected in *Ae. aegypti* Aag2 cell line, but not in another *Ae. aegypti* cell line, Aa20. Because Aag2 cells are one of the commonly used mosquito cells, and a persistent infection with CFAV could affect the cells’ biology and response to other infections, we attempted to remove it from Aag2 cells by RNAi using CFAV *NS5*-specific dsRNA. After continuous transfection of dsRNA into the cells for five passages, CFAV RNA was hardly detectable in the treated cells. Unfortunately, once the dsRNA application was stopped, CFAV came back as soon as one passage and was completely restored to its original levels in three passages. We also found that CFAV significantly promoted the replication of DENV in mosquito cells, particularly if cells were already infected with CFAV prior to DENV infection. Conversely, DENV was also found to moderately enhance the replication of CFAV in *Ae. aegypti* cells.

RNASEK is a protein with two putative transmembrane regions that is highly conserved across vertebrates and invertebrates but its function is largely unknown^34, 35^. A recent study demonstrated that RNASEK promotes the uptake of viruses that enter host cell via clathrin-mediated endocytic route (e.g. flaviviruses, alphaviruses, bunyaviruses, and orthomyxoviruses) but not those that fuse at the cell membrane (e.g. picornavirus and paramyxovirus)^16^. Furthermore, RNASEK was shown to be associated with vATPase and required for the function of vATPase throughout the cells^17^, and depletion of either of the genes led to increase in the pH of endosomes. We also found that silencing RNASEK resulted in reduction in DENV replication in mosquito cells, which corroborates with previous studies with other viruses. Interestingly, our results showed that RNASEK expression was significantly increased in CFAV-infected mosquito cells, which may explain the enhancement of DENV infection in these cells.

With more deep sequencing data becoming available from insects and cell lines showing the presence of a variety of undetected viruses that have established persistent infections, it might be rare that we find a cell line or an insect lab colony that is completely free of any virus. While these cell lines and insect colonies continue to be useful as tools in laboratories, depending on the experimental design and aims of the study, caution should perhaps be undertaken in interpretation of data.

## Electronic supplementary material

Supplementary information

## References

[1] Roby, J., Hall, R. A. & Khromykh, A. A. In *Molecular virology and control of flaviviruses* (ed. P-Y. Shi) 21–50 (Caister Academic Press, 2012).

[2] Halstead SB (2008). Dengue virus-mosquito interactions. Annu Rev Entomol.

[3] Stollar V, Thomas VL (1975). An agent in the *Aedes aegypt*i cell line (Peleg) which causes fusion of *Aedes albopictus* cells. Virology.

[4] Cook S (2006). Isolation of a new strain of the flavivirus cell fusing agent virus in a natural mosquito population from Puerto Rico. J Gen Virol.

[5] Yamanaka A, Thongrungkiat S, Ramasoota P, Konishi E (2013). Genetic and evolutionary analysis of cell-fusing agent virus based on Thai strains isolated in 2008 and 2012. Infect Genet Evol.

[6] Crochu S (2004). Sequences of flavivirus-related RNA viruses persist in DNA form integrated in the genome of *Aedes* spp. mosquitoes. J Gen Virol.

[7] Salas-Benito J, De Nova-Ocampo M (2015). Viral interference and persistence in mosquito-borne flaviviruses. J Immunol Res.

[8] Kean J (2015). Fighting arbovirus transmission: Natural and engineered control of vector competence in *Aedes* mosquitoes. Insects.

[9] Pudney M, Varma M, Leake C (1979). Establishment of cell lines from larvae of culicine (*Aedes* species) and anopheline mosquitoes. TCA Mannual.

[10] O’Brien CA (2015). Viral RNA intermediates as targets for detection and discovery of novel and emerging mosquito-borne viruses. PLoS Negl Trop Dis.

[11] Hussain M, Frentiu FD, Moreira LA, O’Neill SL, Asgari S (2011). *Wolbachia* utilizes host microRNAs to manipulate host gene expression and facilitate colonization of the dengue vector *Aedes aegypti*. Proc Natl Acad Sci USA.

[12] Scott JC (2010). Comparison of Dengue virus type 2-specific small RNAs from RNA interference-competent and –incompetent mosquito cells. PLoS Negl Trop Dis.

[13] Souza-Neto JA, Sim S, Dimopoulos G (2009). An evolutionary conserved function of the JAK-STAT pathway in anti-dengue defense. Proc Natl Acad Sci USA.

[14] Pan X (2012). *Wolbachia* induces reactive oxygen species (ROS)-dependent activation of the Toll pathway to control dengue virus in the mosquito *Aedes aegypti*. Proc Natl Acad Sci USA.

[15] Luplertlop N (2012). Induction of a peptide with activity against a broad spectrum of pathogens in the *Aedes aegypti* salivary gland, following infection with dengue virus. PLoS Pathog.

[16] Hackett BA (2015). RNASEK is required for internalization of diverse acid-dependent viruses. Proc Natl Acad Sci USA.

[17] Perreira JM (2015). RNASEK is a V-ATPase-associated factor required for endocytosis and the replication of rhinovirus, influenza A Virus, and dengue virus. Cell Rep.

[18] Xi Z, Ramirez JL, Dimopoulos G (2008). The *Aedes aegypti* Toll pathway controls dengue virus infection. PLoS Pathog.

[19] Crabtree MB, Sang RC, Stollar V, Dunster LM, Miller BR (2003). Genetic and phenotypic characterization of the newly described insect flavivirus, Kamiti River virus. Arch Virol.

[20] Crabtree MB, Nga PT, Miller BR (2009). Isolation and characterization of a new mosquito flavivirus, Quang Binh virus, from Vietnam. Arch Virol.

[21] Hoshino K (2007). Genetic characterization of a new insect flavivirus isolated from *Culex pipiens* mosquito in Japan. Virology.

[22] Cook S (2009). Isolation of a novel species of flavivirus and a new strain of *Culex* flavivirus (*Flaviviridae*) from a natural mosquito population in Uganda. J Gen Virol.

[23] Junglen S (2009). A new favivirus and a new vector: characterization of a novel flavivirus isolated from Uranotaenia mosquitoes from a tropical rain forest. J Virol.

[24] Bolling BG, Eisen L, Moore CG, Blair CD (2011). Insect-specific flaviviruses from *Culex* mosquitoes in Colorado, with evidence of vertical transmission. Am J Trop Med Hyg.

[25] Calzolari M (2012). Detection of mosquito-only flaviviruses in Europe. J Gen Virol.

[26] Nasar F, Erasmus JH, Haddow AD, Tesh RB, Weaver SC (2015). Eilat virus induces both homologous and heterologous interference. Virology.

[27] Hobson-Peters J (2013). A new insect-specific flavivirus from northern Australia suppresses replication of West Nile virus and Murray Valley encephalitis virus in co-infected mosquito cells. PLoS ONE.

[28] Bolling BG, Olea-Popelka FJ, Eisen L, Moore CG, Blair CD (2012). Transmission dynamics of an insect-specific flavivirus in a naturally infected *Culex pipiens* laboratory colony and effects of co-infection on vector competence for West Nile virus. Virology.

[29] Bolling BG, Weaver SC, Tesh RB, Vasilakis N (2015). Insect-specific virus discovery: significance for the arbovirus community. Viruses.

[30] Kent RJ, Crabtree MB, Miller BR (2010). Transmission of West Nile virus by *Culex quinquefasciatus* Say infected with *Culex* flavivirus Izabal. Plos Neglect. Trop. Dis.

[31] Espinoza-Gómez F (2011). Detection of sequences from a potentially novel strain of cell fusing agent virus in Mexican *Stegomyia* (*Aedes*) *aegypti* mosquitoes. Arch Virol.

[32] Kihara Y (2007). Rapid determination of viral RNA sequences in mosquitoes collected in the field. J Virol Methods.

[33] Cammisa-Parks H, Cisar L, Kane A, Stollar V (1992). The complete nucleotide sequence of cell fusing agent (CFA): homology between the nonstructural proteins encoded by CFA and the nonstructural proteins encoded by arthropod-borne flaviviruses. Virology.

[34] Economopoulou MA, Fragoulis EG, Sideris DC (2007). Molecular cloning and characterization of the human RNase kappa, an ortholog of Cc RNase. Nucleic Acids Res.

[35] Kiritsi MN, Fragoulis EG, Sideris DC (2012). Essential cysteine residues for human RNase k catalytic activity. FEBS J.

