# Supplementary material for: Cell fusing agent virus and dengue virus mutually interact in Aedes aegypti cell lines
Source: Sci Rep. 2017 Jul 31;7:6935. doi: 10.1038/s41598-017-07279-5 (PMC5537255; doi:10.1038/s41598-017-07279-5)
Supplement: Supplementary file 1 — Supplementary information [file 41598_2017_7279_MOESM1_ESM.pdf]

Title: Cell fusing agent virus and dengue virus mutually interact in *Aedes aegypti* cell lines

Guangmei Zhang, Sultan Asad, Alexander A Khromykh, and Sassan Asgari

To comply with the digital image and integrity policies of *Scientific Reports*, in this supplementary file, we present the available original gel images of cropped gel images displayed in the manuscript. Where relevant samples had been run with other samples in the gel, they have been labelled, but not the rest of the samples.

Fig. 1

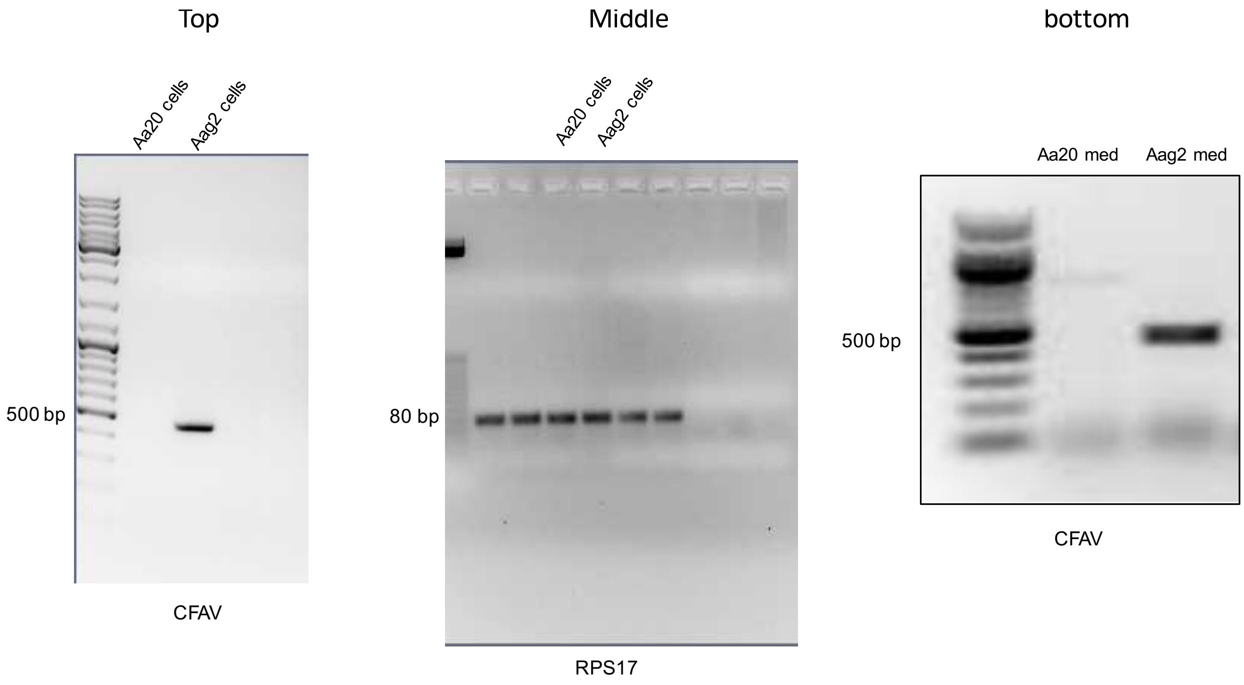

Fig. 2

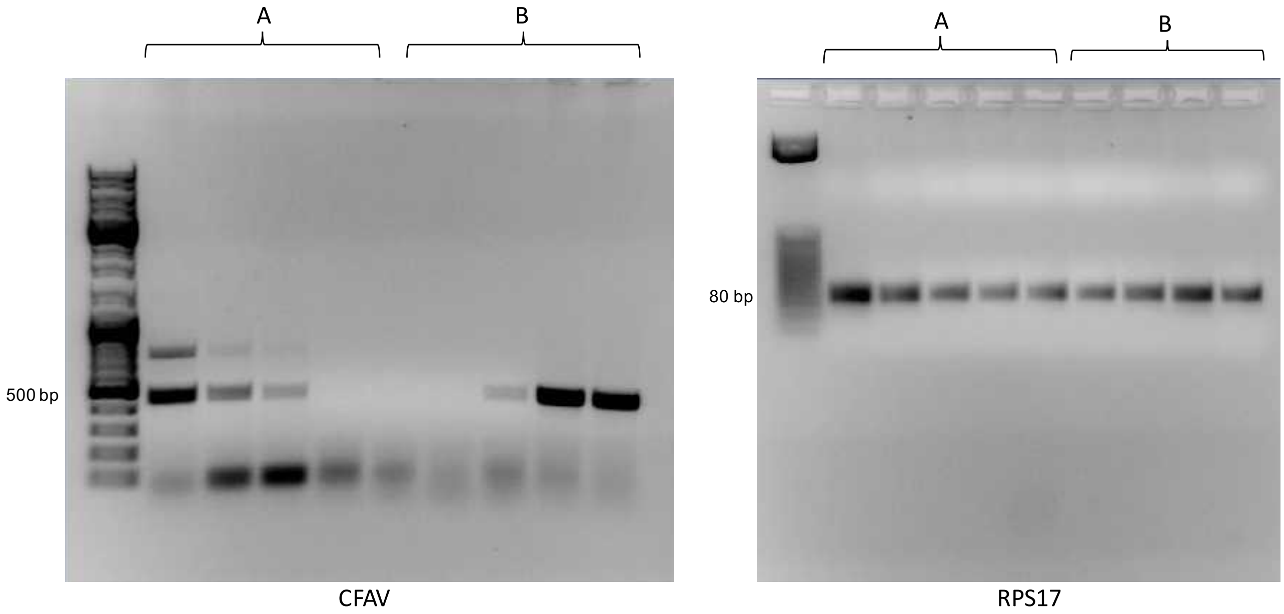
A
B
500 bp
CFAV
A
B
80 bp
RPS17

Fig. 3A

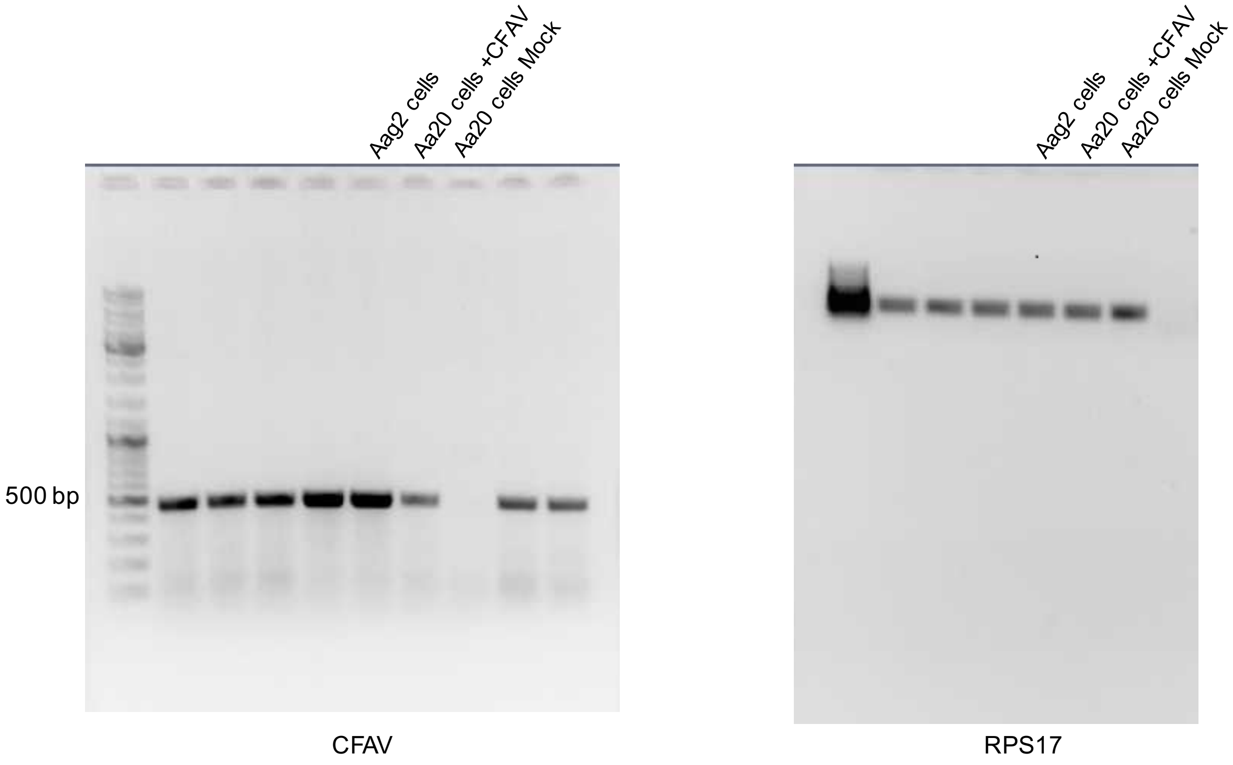
Aag2 cells
Aa20 cells +CFAV
Aa20 cells Mock
500 bp
CFAV
Aag2 cells
Aa20 cells +CFAV
Aa20 cells Mock
RPS17

Fig. 4A and B

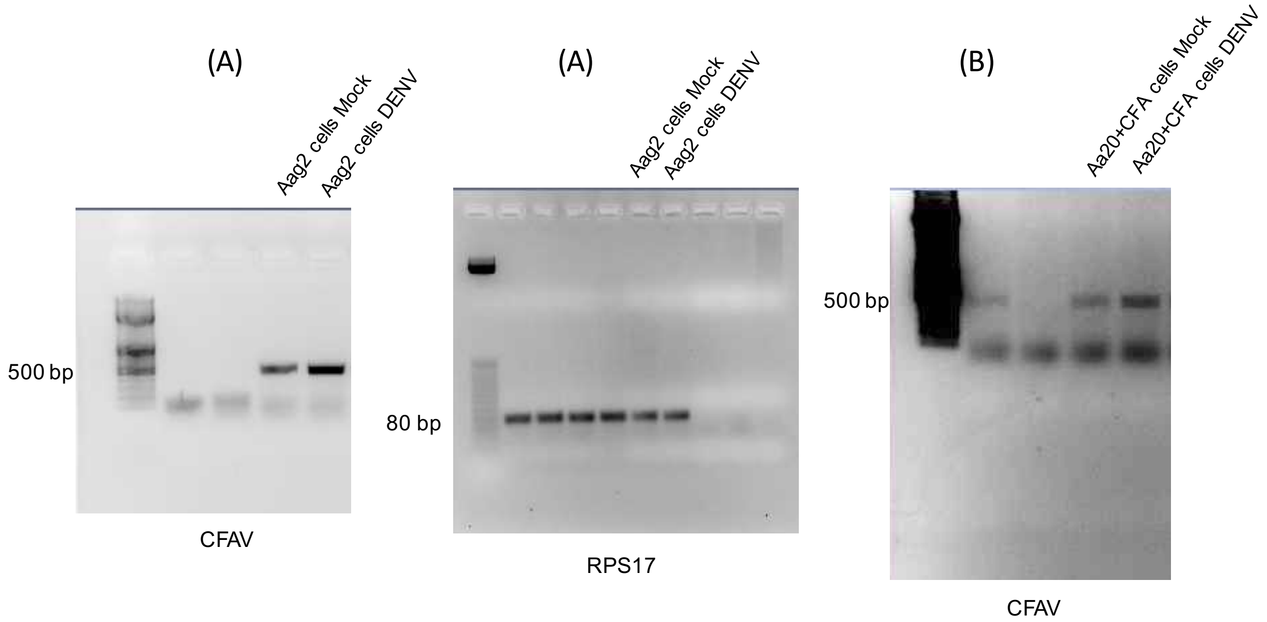
(A)
Aag2 cells Mock
Aag2 cells DENV
500 bp
CFAV
(A)
Aag2 cells Mock
Aag2 cells DENV
80 bp
RPS17
(B)
Aa20+CFA cells Mock
Aa20+CFA cells DENV
500 bp
CFAV
